# Promoting Employee Green Behavior Through the Person-Organization Fit: The Moderating Effect of Psychological Distance

**DOI:** 10.3389/fpsyg.2020.568385

**Published:** 2020-10-09

**Authors:** Lingyun Mi, Yuhuan Sun, Xiaoli Gan, Hang Yang, Tao Lv, Ke Shang, Yaning Qiao, Zhiping Jiang

**Affiliations:** ^1^School of Economics and Management, China University of Mining and Technology, Xuzhou, China; ^2^School of Mechanics and Civil Engineering, China University of Mining and Technology, Xuzhou, China

**Keywords:** employee green behavior, psychological distance, person-organization fit, values fit, needs-supplies fit, demands-abilities fit, structural equation model

## Abstract

The importance of employee green behavior (EGB) to an enterprise’s green development goal is increasingly emphasized in many industries. However, to date promoting EGB through interaction, namely between individuals and organizations, has not been a central concern. Therefore, from the perspective of the person-organization fit, this study considers the psychological distance between employees and the organization as a moderating variable, exploring the mechanisms of values fit, needs-supplies fit, and demands-abilities fit on green behaviors as within and outside the scope of employee responsibility. After collecting the results of questionnaires from 412 employees, our hypotheses were tested using the Structural Equation Model (SEM). The results show that (1) person-organization fit can effectively promote EGB in the workplace. However, different types of person-organization fit have different influencing paths and effect-strengths on employees’ task-related green behavior and proactive green behavior. (2) Values fit has the greatest incentive effect on EGB, followed by demands-abilities fit, while needs-supplies fit promotes only eco-helping behavior. (3) Psychological distance has a significant moderating effect on the relationship between the person-organization fit and EGB. The effect of person-organization fit on EGB is enhanced when employees are close with less emotional distance, while the effect is weakened in the case of close expectation distance. Finally, this study provides suggestions for enterprise managers providing ways to motivate EGB through the selection and allocation of human resources.

## Introduction

Global warming, water pollution, air pollution, and other environmental issues are becoming increasingly serious, making environmental sustainability a high concern (He et al., 2016; Bansal and Song, 2017). Research shows that social production and enterprises are a decisive force for sustainable development (Figueres et al., 2018). An increasing number of enterprises are implementing corporate social responsibility and/or sustainable development strategies. However, research shows that these strategies can only reduce the environmental impacts of organizations to a certain extent (King et al., 2005), and the response of employees to these strategies is a crucial boundary condition (Davis et al., 2011). The implementation of corporate green measures largely depends on the cooperation and participation of employees (Zhang et al., 2013). Therefore, employee green behavior (EGB) is key to promoting the green development of an enterprise (Haugh and Talwar, 2010). EGB refers to all the environmentally sustainable behaviors implemented by employees in the workplace (Ones and Dilchert, 2012). On the one hand, EGB can achieve a competitive advantage for the company (Brio et al., 2007), improve the company’s environmental performance, and earn them an environmental reputation (Paillé et al., 2014). At the same time, it can also improve the company’s market orientation, save costs, and reduce resource consumption (Chen et al., 2015). On the other hand, for employees, implementing green behavior can improve their work motivation (Osbaldiston and Sheldon, 2003), increase job satisfaction (Norton et al., 2014), and promote their career development (Bauer and Aiman-Smith, 1996).

Existing studies on EGB mainly focus on individual factors and the situational factors of employees (Norton et al., 2015). Among them, individual factors mainly include personality traits (Kim et al., 2017), affections (Bissing-Olson et al., 2013), attitudes, values, personal norms (Liu et al., 2018; Gkargkavouzi et al., 2019), perceived behavioral control (Greaves et al., 2013), and environmental knowledge (Liobikienė and Poškus, 2019). Situational factors mainly include organizational support (Lamm et al., 2013), leadership style (Graves et al., 2013; Mi et al., 2019), green atmosphere (Norton et al., 2014), green human resource management practices (Dumont et al., 2017), and corporate social responsibility (Tian and Robertson, 2019). These studies provided a basis for our understanding of EGB. However, the role of the interaction between individuals and organizations in terms of EGB has not yet received wide attention.

Individual employees exist in interdependent organizational situations. Their behaviors will not only be affected by personal factors and their specific organizational context but also by their interaction with other individuals and their organizations (i.e., person-organization fit) (Afsar and Badir, 2016). Person-organization fit refers to the compatibility between individuals and their organizations. This is an important factor in predicting individuals’ attitudes and behaviors (Kristof, 1996). When the individual’s values, capacity, and demand and the organizational values, job requirements (specification), as well as remuneration system fit well, individuals will experience higher job satisfaction (Jin et al., 2018; Roczniewska et al., 2018), organizational commitment (Bahat, 2020), lower work-related pressure (Gould-Williams et al., 2015), and lower turnover intention (Memon et al., 2018). At the same time, they will also implement more pro-organizational behaviors, such as performance behaviors (Goodman and Svyantek, 1999), organizational citizenship behaviors (Kristof-Brown et al., 2005; Hoffman and Woehr, 2006), and innovative behaviors (Afsar et al., 2015). This paper is interested in whether the person-organization fit may motivate employees to implement green behaviors. If the person-organization fit can effectively promote EGB, it will provide a new path with high potential to promote workplace sustainability.

Recently, research on psychological distance has begun to emerge in the field of environmental science, aiming to better promote public environmental behavior (Li et al., 2020). Ryoo et al. (2017) use psychological distance to explain the failure of the public to implement environmental protection. Yu et al. (2017) explore the relationship between the decrease in psychological distance related to climate change and loyalty to green products from the perspective of environmental sustainability. Later, Chen and Li (2018) introduced psychological distance into the study of organizational behavior and proposed the concept of “employee-organizational psychological distance” (EOPD), used to describe employees’ subjective judgment of the distance between themselves and organizations. Chen and Li (2018) point out that employees’ perception of psychological distance affects their emotional experience and behavior in the organization. For example, when the psychological distance between employees and organizations is relatively close, several positive employee characteristics will be activated and often manifest as increased occupational mental health. Conversely, when the psychological distance is greater, employees will pay more attention to the negative impact of working, and their mental health levels will also be negatively affected (Liu et al., 2020). The application of psychological distance in the field of environmental protection and organizational behavior provides new ideas and theoretical methods for solving current environmental behavior problems. Therefore, this study attempts to introduce psychological distance as a moderating variable to further analyze the internal influence mechanism of person-organization fit on EGB. In this study, psychological distance refers to employees’ subjective judgment as well as evaluation of the distance between themselves and organizations, which is used to describe the strength of the psychological connection between employees and organizations.

This study provides the contributions to the field: first, it expands the person-organization fit theory, exploring the influence of values fit, needs-supplies fit, and demands-abilities fit on EGB. At the same time, it extends the antecedent’s study of EGB from the individual level to the interaction level between individuals and organizations, providing a new perspective for understanding and predicting EGB in the workplace. Second, this study introduces psychological distance into the study of EGB, dividing it into two dimensions, emotional distance, and expectation distance, thus examining the moderating effect of psychological distance. This is an important supplement to the existing literature on the topic. Finally, this study provides powerful suggestions for how to motivate EGB through the selection and allocation of human resources and thus is conducive to promoting the greening of human resource management. Moreover, this study responds to the dynamic nature of the mechanism of EGB, which cannot be solved by mandatory regulations and technological progress.

The paper is organized as follows: in section “Theoretical Basis and Hypothesis,” research hypotheses are proposed after a literature review. Section “Research Methodology” then describes the research method and data collection. Section “Results” presents the data analysis results obtained from empirical tests, and the discussion takes place in Section “Discussion.” Finally, the conclusions, implications, and suggestions for future research are discussed in the last sections.

## Theoretical Basis and Hypothesis

### Employee Green Behavior

Green behavior refers to any behavior that is beneficial to the environment or minimizes harm to the environment (Steg and Vlek, 2009). With the implementation of enterprise sustainable development strategy, increasing attention has been paid to the green behavior of employees in the workplace (Norton et al., 2015; Wang et al., 2018). EGB refers to all environmentally sustainable behaviors implemented by employees in the workplace (Ones and Dilchert, 2012). According to the autonomous standards of behavior (organizational requirements and individual self-determination), EGB includes two aspects: task-related green behavior implemented within employee responsibilities and proactive green behavior implemented outside of employee responsibilities (Bissing-Olson et al., 2013). Task-related green behavior refers to the green behavior enacted by employees to complete the core work tasks required by the organization (such as environmental protection responsibilities stipulated in the performance of duties, compliance with environmental standards, etc.). Proactive green behavior refers to discretionary and environmentally friendly behavior that is not explicitly recognized by the formal reward system (Boiral, 2009; Bissing-Olson et al., 2013) (such as double-sided printing, reminding colleagues to save energy, etc.).

Employee proactive green behavior can not only directly contribute to the environmental performance of enterprises but also help fill the environmental gap that the enterprises’ formal rules and regulations do not pay attention to Alt and Spitzeck (2016) and Raineri and Paillé (2016). This kind of proactive green behavior is also known as organizational citizenship behavior for the environment (OCBE) (Daily et al., 2009). Boiral and Paille (2012) divided it into three dimensions: eco-initiatives behavior, eco-civic engagement behavior, and eco-helping behavior. Among them, eco-initiatives behavior refers to employees’ initiative to promote the enterprise’s environmental protection practice, which indicates the employees’ intrinsic environmental protection motivation. Eco-civic engagement behavior refers to employees’ voluntary participation in the organizations’ environmental projects and activities. Eco-helping behavior refers to helping and encouraging colleagues to pay more attention to environmental issues. The effectiveness of this three-dimensional division is confirmed by Terrier et al. (2016) and Boiral et al. (2018). Thus, according to the research of Boiral and Paille (2012) and Bissing-Olson et al. (2013), we divide EGB into four dimensions: task-related green behavior, eco-initiatives behavior, eco-civic engagement behavior, and eco-helping behavior.

### The Influence of Person-Organization Fit on EGB

The concept of person-organization fit is derived from interactive psychology, which is developed from the person-environment fit theory. It is generally defined as the adaptability of individuals and organizational environments, and it is interpreted as the common basic characteristics between individuals and the organizational environment or the ability of the two to meet the other’s needs (Chatman, 1989). The well-known A-S-A (attraction-selection-attrition) model proposed by Schneider (1987) explains the formation mechanism of the fit between individuals and organizational environments. Schneider believes that people are attracted to organizations with personality characteristics similar to their own, enter organizations through organizational-selections and self-selections, and decide to stay or resign during the process of organizational socialization. The A-S-A model emphasizes that personal goals and values conform to the values, goals, and personality traits of the organization’s founders. Subsequently, Kristof (1996) divided the concept of person-organization fit into two dimensions: similarity fit and complementary fit. Similarity fit refers to the degree of consistency between the basic characteristics of individuals (values, personality, goals, and attitudes) and those of organizations (values, atmosphere, goals, and norms). Complementary fit means that the needs of organizations (individuals) are satisfied by the supply of individuals (organizations). Based on Kristof’s (1996) classification, Cable and DeRue (2002) believe that, in addition to similarity fit, the complementary fit should be subdivided into needs-supplies fit and demands-abilities fit. Needs-supplies fit refers to the degree to which the supply of work can meet individual needs. Demands-abilities fit refers to the degree of fit between employees’ knowledge, skills, ability, and job requirements. Moreover, their research proves that the three kinds of fit perceptions are independent and the three dimensions point to different result variables, respectively. The result of factor analysis also confirms that the conceptual model of three-dimensional fit is indeed superior to the two-dimensional one by Kristof (1996). Therefore, our study uses the three-dimensional fit model of Cable and DeRue (2002) for reference to divide the person-organization fit into values fit, needs-supplies fit, and demands-abilities fit.

Chatman (1991) argues that values fit is the most important factor affecting the person-organization fit. He defines values fit as the consistency between employee values and organizational values. Individual values, which represent a series of basic beliefs of individuals, are the reference and selection criteria when people measure their behavior and goals, and the standard for individuals to judge right or wrong, beauty or ugliness, good or evil (Robbins and Judge, 2009). Organizational values are the core and soul of organizational culture (Hofstede et al., 1990), which refers to the normative beliefs shared by the members of an organization. These beliefs reflect the pursuit of goals that the organization considers to be the most valuable and that have become the code of conduct and norms for the members of the organization (O’Reilly et al., 1991). Previous studies have found that a good organizational culture can promote individuals’ extra-role behavior (Goodman and Svyantek, 1999). When employees are in harmony with the values of the organization, they experience a sense of belonging to and identity in the organization, which, in turn, results in them forming favorable attitudes and behaviors toward the organization (Saks and Ashforth, 1997; Cable and DeRue, 2002; Gould-Williams et al., 2015). According to the findings of Cable and DeRue (2002) in a study of 185 MBA graduates from Southeast University, 187 managers from 143 different organizations, and 135 supervisors or peers, a higher values fit between individuals and organizations can effectively reduce employees’ turnover intention and generate more extra-role behaviors such as organizational citizenship behavior (OCB). Later, Gould-Williams et al. (2015) conducted a study with Egyptian public sector managers and confirmed that when employees believe their values and goals fit those of the organization, they are more likely to participate in OCB. EGB in the workplace, whether it is the green behavior required by the task or proactive green behavior outside of employee responsibilities, is of vital importance. It is valuable to the organization’s implementation of environmental regulations, improvement of environmental performance, development of green innovation, and establishment of an environmental-protection-centered reputation. In particular, employee proactive green behavior outside organizational roles, also known as OCBE, is a special type of OCB. Therefore, it is reasonable to infer that when employees’ values fit those of the organization, it will also promote EGB. Therefore, we propose H1:

H1: Values fit positively affects EGB.
H1a: Values fit positively affects task-related green behavior.
H1b: Values fit positively affects eco-initiatives behavior.
H1c: Values fit positively affects eco-civic engagement behavior.
H1d: Values fit positively affects eco-helping behavior.

Needs-supplies fit refers to the consistency between the needs of employees and the rewards (such as wages, benefits, training, promotion, etc.) obtained from their contributions at work (Cable and DeRue, 2002). Needs-supplies fit is used to guide employees’ behavior according to several extensive theories such as Herzberg’s motivator-hygiene theory (dual-factor theory) (Herzberg, 1966), Maslow’s hierarchy of needs (Maslow, 1954), and the expectancy theory of motivation (Vroom, 1964). In these theoretical models, organizational members try to maximize their benefits and minimize their costs. Employees plunge time and energy into their careers to generate the rewards they need in financial (e.g., pay level), social (e.g., good peers), or psychological (e.g., power over others) aspects. From the perspective of employees, the needs-supplies fit is probably the most important type of fit (Cable and DeRue, 2002). Individuals’ attitudes toward the organization depend on the degree of consistency between their needs and the benefits and rewards provided by the organization to meet those needs. When the needs of employees are met, they experience a positive work attitude that promotes better behavior (Cable and Edwards, 2004; Kristof-Brown et al., 2005). Cable and DeRue (2002) show that needs-supplies fit has a significant positive impact on employees’ job satisfaction, career satisfaction, and career commitment. A meta-analysis by Kristof-Brown et al. (2005) on 172 existing studies about person-environment fit also finds that needs-supplies fit was positively correlated with job satisfaction. Other studies confirm that employees’ job satisfaction (Paillé and Boiral, 2013) can promote OCBE. In summary, we propose that when the individual needs of employees in the organization are met, higher job satisfaction will be generated, and employees will be more willing to implement green behaviors to benefit the organization. Therefore, we propose H2:

H2: Needs-supplies fit positively affects EGB.
H2a: Needs-supplies fit positively affects task-related green behavior.
H2b: Needs-supplies fit positively affects eco-initiatives behavior.
H2c: Needs-supplies fit positively affects eco-civic engagement behavior.
H2d: Needs-supplies fit positively affects eco-helping behavior.

Demands-abilities fit is the main measure of person-job fit (Kristof-Brown, 2000). A basic principle of industrial psychology is that a high degree of consistency between personal abilities and job requirements leads to higher job performance. On the one hand, if individuals’ ability level is lower than the job requirements, the efficiency of the work process, and the quality of the work results will be reduced. The low performance will cause employees to feel frustrated and affect their self-esteem, thereby reducing job satisfaction. On the other hand, if individuals’ ability level is much higher than the job requirements, they may feel that they are not fully utilized, and will invest less in their career, which will harm the organization (Cable and DeRue, 2002). Therefore, the fit of employees’ abilities and job requirements has always been the focus of research (Kristof-Brown, 2000; Venkatesh et al., 2017). When an employee first approaches a job, the demands-abilities fit predicts the organization’s attractiveness to them and determines whether the employee will take the job or not (Carless, 2005). After entering the organization, the fit of personal ability and job requirements becomes an important predictor of employees’ performance. This has been confirmed by many studies (Ferris and Judge, 1991; Scroggins, 2008; Lin et al., 2014). Moreover, a high demands-abilities fit will also increase employees’ job satisfaction (Nguyen and Borteyrou, 2016). In addition, Afsar et al. (2015) researched multi-source data from 459 employees and their supervisors and found that demands-abilities fit can also positively promote employees’ innovative behaviors. In summary, if task-related green behavior is a job requirement, the demands-abilities fit will have a positive impact on it. Proactive green behavior is behavior outside of the employees’ role; therefore, we draw similarities between innovative behavior and proactive green behavior as both are individual and self-determined. Although there are no hard-and-fast requirements for rules and regulations, these are also constantly advocated and promoted by the organization. Therefore, we propose H3:

H3: Demands-abilities fit positively affects EGB.
H3a: Demands-abilities fit positively affects task-related green behavior.
H3b: Demands-abilities fit positively affects eco-initiatives behavior.
H3c: Demands-abilities fit positively affects eco-civic engagement behavior.
H3d: Demands-abilities fit positively affects eco-helping behavior.

### The Moderating Effect of Psychological Distance

In the natural sciences, “distance” refers to the length of time or space between specific objects. “Psychological distance” originates from western aesthetics. The concept was first proposed by the Swiss psychologist Bullough (1912). He focuses on visual art and suggests that psychological distance refers to the separation of the actual interests between the viewer of the artwork and the artwork itself, rather than the distance in time or space (Bullough, 1912). Later, scholars studied psychological distance from different perspectives. Researchers concerned with the perspective of information flow believed that psychological distance is a negative factor for the flow of information between the host country market and multinational companies (Brewer, 2007), including differences in religion, lifestyle, business practices, language, and culture (Madsen, 1989). Researchers with a subjective perception perspective believe that psychological distance is not a simple collection of external environments but is closely related to the perception of individuals. It is the perception of the differences between the management of multinational companies and other countries. Cultural background, educational level, international experience, language ability, and values all affect this subjective perception (Evans and Mavondo, 2002; Prime et al., 2009). The construal level theory (CLT) proposes that psychological distance refers to an individual’s perception of distance, such as distance in time and space, affinities or estrangements in social relations, and the probability of occurrence of certain events or behaviors based on his or her own direct experience as a reference point (Trope et al., 2007). Most of the concepts of psychological distance in these studies were used in international business and cross-cultural management (Håkanson, 2014; Ciszewska-Mlinarič and Tra̧pczyński, 2016), which were later introduced into the study of interpersonal communication and social relations (Huang, 2015). Recently, to better describe the relationship between employees and organizations, Chen and Li (2018) introduced psychological distance into the study of organizational behavior, and the concept of EOPD was proposed. EOPD can be used to describe the level of perceived correspondence or interaction between employees and organizations, which is a direct reflection of the relationship between employees and organizations. In this study, we define psychological distances as employees’ subjective judgment as well as evaluation of the distance between themselves and organizations. It is used to describe the strength of the psychological connection between employees and organizations.

Because of different research perspectives and research fields of psychological distance, understandings of psychological distance are not the same. CLT believes that psychological distance includes time, space, social distance, and probability (Trope et al., 2007). Xiao and Nie (2018) divide the psychological distance of employees into four dimensions: expectation distance, power distance, professional background distance, and regional culture distance. Chen and Li (2018) divide the psychological distance into six dimensions: experiential distance, behavioral distance, emotional distance, cognitive distance, spatial-temporal distance, and objective social distance. These dimensional divisions of psychological distance include the psychological relationship of the internal driving force and the realistic relationship of the external driving force. As psychological distance assesses the distance between employees’ perception of the relationship between themselves and the organization, this is also a manifestation of social exchange relationships. In social exchange theory, social exchange relations are usually divided into the social exchange and economic exchange. The former emphasizes emotional relations, while the latter focuses on interest relations (Shore et al., 2006). According to the above literature, we divide the psychological distance between individuals and organizations into emotional distance and expectation distance only from the perspective of the psychological relationship of the internal driving force. Emotional distance refers to employees’ emotional judgment of the partnership formed in their daily interactions with the organization. Expectation distance refers to the degree of acceptance of the gap between the employees’ actual gains in the organization and their expected benefits as per their interests. The former emphasizes the degree of distance between employees and the organization in their emotional relationship, while the latter emphasizes this between employees and the organization in their professional relationship.

Studies have shown that the psychological distance (time, space, social distance, and probability) of different dimensions can guide behavior through mental construal (Trope et al., 2007). In terms of environmental protection, Spence et al. (2012) use CLT to measure British consumers’ psychological distance from climate change and their willingness to use eco-friendly energy. They find that consumers with a lower level of psychological distance show higher environmental concerns and willingness to save energy. Yu et al. (2017) explore the relationship between the shrinking of psychological distance associated with people’s relationship to climate change and their loyalty to green products. Their study supports the idea that psychological distance can affect people’s willingness to protect the environment through the intermediary role of environmental ethics and social responsibility. Similarly, in organizational practice, employees are often self-centered when they perceive various information (salary, promotion space, colleagues’ relationship, etc.). After integrating this information, employees form a subjective perception and emotional experience of their relationship distance with the organization (Li and Chen, 2019), which may be manifested as attraction or rejection (Agnew et al., 2004).

Liu et al. (2020) found that psychological distance between employees and the organization may positively moderate the relationship between work hours and employees’ occupational mental health, and a “close” employee-organizational psychological distance may alleviate the pressure of work hours, thus helping to maintain high-quality occupational mental health. Similarly, when employees and organizations are in a “distant” relationship, it may weaken employees’ sense of identity and belonging to the organization, thereby making it more difficult to implement EGB. On the contrary, individuals have a high level of psychological involvement with organizations that are “closer,” potentially activating several positive psychological variables, one of which is adopting green behavior. Therefore, we speculate that the impact of different types of fits between individuals and organizations on EGB may also be moderated by psychological distance. To better analyze the relationship between person-organization fit and EGB, this study attempts to incorporate psychological distance as a moderating variable into the model of person-organization fit and EGB. Thus, we propose H4 and H5:

H4: Emotional distance will moderate the effect of person-organization fit on EGB, such that the effect will be greater when the emotional distance is close rather than distant.
H5: Expectation distance will moderate the effect of person-organization fit on EGB, such that the effect will be greater when expectation distance is close rather than distant.

**FIGURE 1:**
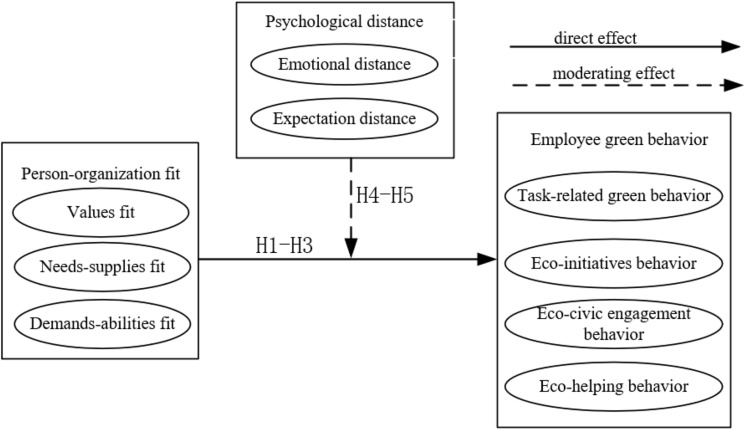
Conceptual model.

The theoretical model of this study is shown in Figure 1.

## Research Methodology

### Survey Sample

We randomly selected and contacted human resource managers from six different industries in an MBA class at a Chinese university, and informed them of our research purpose, data collection procedures, and data confidentiality. With their support, we obtained the email addresses of the employees of their enterprises. The anonymous questionnaire survey was conducted online in February 2020. It contained three sections of measurements: person-organization fit, psychological distance, and EGB. E-mails were sent to 548 employees. They were informed that the study would be conducted anonymously and would not link anyone’s name or other private information with the final questionnaire data. This was ensured to alleviate employee concerns. A total of 467 questionnaires were received in this study. After eliminating 55, which had incomplete or casual answers, 412 valid questionnaires were obtained. The sample size meets the SEM indicator requirements set by Mueller (1997) (the ratio of the sample size to the number of measured items is at least between 10:1 and 15:1). The structural characteristics of the samples are shown in Table 1.

**TABLE 1:** Sample demographic characteristics (*N* = 412).

Variable	Category	Number	Percentage
Gender	Male	209	50.7%
	Female	203	49.3%
Age	<20	30	7.3%
	20–30	221	53.6%
	31–40	99	24.0%
	41–50	51	12.4%
	>50	11	2.7%
Education	Below junior high school	38	9.2%
	High school, technical secondary school or technical school	47	11.4%
	Bachelor’s degree	259	62.9%
	Graduate degree	68	16.5%
Profession	General workers	278	67.5%
	Junior managers	75	18.2%
	Middle managers	52	12.6%
	Senior managers	7	1.7%
Monthly disposable income	Below 3,000 CNY	97	23.5%
	3,000–5,000 CNY	112	27.2%
	5,000–10,000 CNY	128	31.1%
	10,000–20,000 CNY	49	11.9%
	20,000–50,000 CNY	16	3.9%
	More than 50,000 CNY	10	2.4%

### Variable Measurement

The measures of the constructs in this study were based on established scales. All items used a five-point Likert scale ranging from 1 = “strongly disagree”/“never” to 5 = “strongly agree”/“always.” Supplementary Appendix 1 shows the complete questionnaire.

#### Person-Organization Fit

The person-organization fit scale is based on the three-dimensional scale developed by Cable and DeRue (2002). The scale has been used by several scholars and has been proven to be highly credible. Three items assessed values fit (e.g., “The things that I value in life are very similar to the things that my organization values”); three items assessed needs-supplies fit (e.g., “There is a good fit between what my job offers me and what I am looking for in a job”); and three items assessed demands-abilities fit (e.g., “The match is very good between the demands of my job and my personal skills”).

#### Psychological Distance

The psychological distance scale is mainly based on the organization-employee emotional distance scale developed by Chen and Li (2018) and the organization-employee expectation distance scale developed by Nie (2017). Four items assessed emotional distance (e.g., “I will protect organizational interests at the cost of my own interests when necessary”) and two items assessed expectation distance (e.g., “I will work harder only if the return of work meets my expectations”).

#### Employee Green Behavior

The EGB scale is divided into task-related green behavior and proactive green behavior. Task-related green behavior was revised regarding the employee task performance scale (Bachrach et al., 2007). Four items assessed task-related green behavior (e.g., “I can accomplish the environmental protection tasks within my duties competently”). Proactive green behavior was revised based on the scale developed by Boiral and Paille (2012) and Paillé et al. (2016), and localized corrections were made. The revised scale consists of three dimensions (i.e., eco-initiatives behavior, eco-civic engagement behavior, and eco-helping behavior). Three items assessed the eco-initiatives behavior (e.g., “I pay attention to energy conservation and low-carbon travel in my daily work”); three items assessed eco-civic engagement behavior [e.g., “I actively participate in environmental events organized by my company (or department)”]; and five items assessed eco-helping behavior (e.g., “I am willing to spend time reminding my colleagues to pay attention to environmental protection at work”).

### Scale Test

Considering that the questionnaire is filled out by the same object, it may cause a common method bias (Harman, 1976). For this reason, before hypothesis testing, we conducted a common method bias test. The results of the Harman single factor test showed that the contribution rate of the largest factor precipitated is 47.187%, which is lower than the threshold value of 50%, indicating that common method bias was unlikely to be a serious problem in this study (Podsakoff and Mackenzie, 2003).

Then, we conducted a confirmatory factor analysis (CFA) by using the maximum likelihood method with Mplus7.4. We compared four different models: Single model, in which all questions measure the same factor; double-factor model, that is, EGB measures one factor, psychological distance and person-organization fit measure the other one; triple-factor model consists of EGB, psychological distance, and person-organization fit; nine-factor model consists of values fit, needs-supplies fit, demands-abilities fit, task-related green behavior, eco-initiatives behavior, eco-civic engagement behavior, eco-helping behavior, emotional distance, and expectation distance. The summary of model fit indices is presented in Table 2. As shown in Table 2, compared with the other three models, the nine-factor model fits the data best. The fit index was up to the standard (Hu and Bentler, 1998), which means that there was a good distinction between the constructs.

**TABLE 2:** Summary of model fit indices.

Model	χ^2^	DF	χ^2^/DF < 3	CFI >0.9	TLI >0.9	RMSEA <0.08	SRMR <0.08
M1: Single model	3292.615	405	8.130	0.693	0.670	0.132	0.091
M2: Double-factor model	2798.497	404	6.927	0.746	0.726	0.120	0.084
M3: Triple-factor model	1877.531	402	4.670	0.843	0.830	0.094	0.100
M4: Nine-factor model	881.054	369	2.388	0.946	0.936	0.058	0.032

*χ^2^, chi-square statistic; DF, the degree of freedom; CFI, comparative fit index; TLI, Tucker-Lewis index; RMSEA, root mean squared error of approximation; SRMR, standardized root mean squared residual.*

Reliability and validity were tested using SPSS19.0 and Mplus7.4 (see Table 3). The standardized factor loadings range from 0.717 to 0.917 for all items and are greater than the threshold value of 0.6. Cronbach’s α are from 0.706 to 0.921, and composite reliability (CR) values are from 0.712 to 0.921, which are all greater than the recommended value of 0.7 (Hair et al., 2010). The results indicate that the scale has appropriate reliability. The average variance extracted (AVE) are from 0.553 to 0.744 and are all greater than the recommended value of 0.5, which indicates that the scale has an appropriate convergence validity.

**TABLE 3:** Results of reliability and validity tests.

Variable	Item	Estimate	Cronbach’s α	Composite reliability	Convergence validity
				CR	AVE
Task-related green behavior	TRGB 1	0.801	0.885	0.885	0.657
	TRGB 2	0.812			
	TRGB 3	0.815			
	TRGB 4	0.815			
Eco-initiatives behavior	EIB 1	0.777	0.842	0.844	0.643
	EIB 2	0.790			
	EIB 3	0.837			
Eco-civic engagement behavior	ECB 1	0.830	0.877	0.877	0.705
	ECB 2	0.850			
	ECB 3	0.838			
Eco-helping behavior	EHB 1	0.815	0.921	0.921	0.700
	EHB 2	0.859			
	EHB 3	0.822			
	EHB 4	0.854			
	EHB 5	0.833			
Values fit	VF 1	0.760	0.828	0.828	0.616
	VF 2	0.781			
	VF 3	0.812			
Needs-supplies fit	NSF 1	0.813	0.857	0.860	0.672
	NSF 2	0.858			
	NSF 3	0.787			
Demands-abilities fit	DAF 1	0.817	0.851	0.853	0.659
	DAF 2	0.824			
	DAF 3	0.794			
Emotional distance	EMD 1	0.798	0.920	0.921	0.744
	EMD 2	0.843			
	EMD 3	0.917			
	EMD 4	0.887			
Expectation distance	EXD 1	0.770	0.706	0.712	0.553
	EXD 2	0.717			

*TRGB, Task-related green behavior; EIB, Eco-initiatives behavior; ECB, Eco-civic engagement behavior; EHB, Eco-helping behavior; VF, Values fit; NSF, Needs-supplies fit; DAF, Demands-abilities fit; EMD, Emotional distance; EXD, Expectation distance.*

## Results

### Descriptive Analysis and Correlation Analysis

Descriptive statistical analysis is performed to obtain an overall understanding of the data and the correlation between variables is calculated to clarify the intensity of the correlation between each variable. The numbers in the cells of the diagonal line are the square root of AVE. The results show that the square root value of AVE for each latent variable is greater than the correlation of all the remaining constructs in the row and column in which it is located. Therefore, the structure has an appropriate discriminant validity (Fornell and Larcker, 1981). The mean (M), standard deviation (SD), and the Pearson correlation coefficient for all variables are presented in Table 4. There are significant correlations between EGB, person-organization fit, and psychological distance. These results provide the basis for the following hypothesis testing.

**TABLE 4:** Descriptive statistical analysis.

Dim	M	SD	Discriminate Validity								
			1	2	3	4	5	6	7	8	9
TRGB	4.052	0.794	0.811								
EIB	3.971	0.853	0.739**	0.802							
ECB	3.866	0.894	0.682**	0.774**	0.840						
EHB	3.621	0.986	0.610**	0.688**	0.754**	0.837					
VF	3.972	0.749	0.648**	0.615**	0.650**	0.682**	0.785				
NSF	3.846	0.844	0.603**	0.587**	0.624**	0.694**	0.778**	0.820			
DAF	4.006	0.781	0.660**	0.643**	0.612**	0.618**	0.747**	0.754**	0.812		
EMD	3.604	1.048	0.269**	0.248**	0.284**	0.331**	0.296**	0.336**	0.327**	0.863	
EXD	3.678	0.938	0.320**	0.372**	0.392**	0.421**	0.429**	0.463**	0.443**	0.160**	0.744

*N* = 412. **p < 0.01. The numbers in the cells of the diagonal line are the square root of AVE. TRGB, Task-related green behavior; EIB, Eco-initiatives behavior; ECB, Eco-civic engagement behavior; EHB, Eco-helping behavior; VF, Values fit; NSF, Needs-supplies fit; DAF, Demands-abilities fit; EMD, Emotional distance; EXD, Expectation distance.

### Structural Equation Model and Path Analysis

To test our hypotheses, Mplus7.4 was used to verify the complete structural equation model (SEM). According to the fitting indices suggested by Hu and Bentler (1998), the model in this study has a good fitting effect (χ^2^ = 610.575, DF = 231, χ^2^/DF = 2.643 < 3, CFI = 0.951 > 0.9, TLI = 0.941 > 0.9, RMSEA = 0.063 < 0.08, SRMR = 0.030 < 0.08). Table 5 reports the direct effect results of person-organization fit on EGB.

**TABLE 5:** Analysis results of the direct effects.

Path relationship	Estimate	S.E.	Est./S.E.	*P*-Value	Supported
(1)VF →TRGB	0.544	0.224	2.427	0.015*	YES
(2)VF→EIB	0.390	0.226	1.728	0.084	NO
(3)VF→ECB	0.605	0.226	2.681	0.007**	YES
(4)VF→EHB	0.506	0.206	2.463	0.014*	YES
(5)NSF→TRGB	−0.213	0.212	−1.006	0.314	NO
(6)NSF→EIB	−0.105	0.212	−0.496	0.620	NO
(7)NSF→ECB	0.045	0.208	0.216	0.829	NO
(8)NSF →EHB	0.375	0.19	1.973	0.049*	YES
(9)DAF→TRGB	0.467	0.139	3.354	0.001**	YES
(10)DAF→EIB	0.505	0.143	3.526	***	YES
(11)DAF→ECB	0.129	0.142	0.911	0.362	NO
(12)DAF→EHB	−0.082	0.132	−0.625	0.532	NO

*p < 0.05, **p < 0.01, ***p < 0.001. TRGB, Task-related green behavior; EIB, Eco-initiatives behavior; ECB, Eco-civic engagement behavior; EHB, Eco-helping behavior; VF, Values fit; NSF, Needs-supplies fit; DAF, Demands-abilities fit.

**FIGURE 2:**
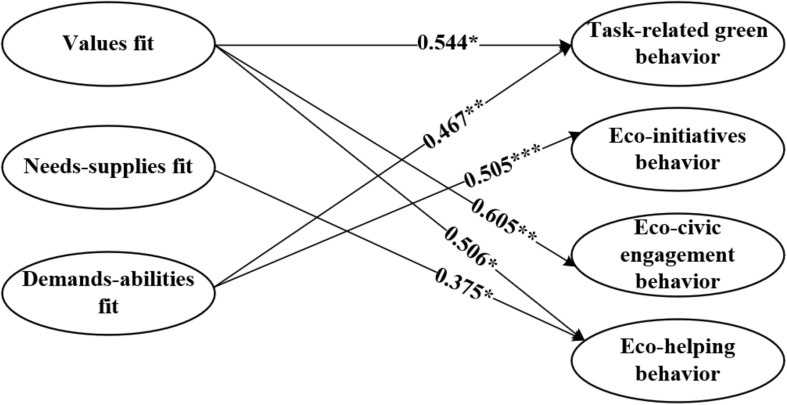
Final results of the structural equation model. *p < 0.05, **p < 0.01, ***p < 0.001.

Table 5 shows that values fit has a significant positive effect on task-related green behavior (β = 0.544, *p* = 0.015 < 0.05), eco-civic engagement behavior (β = 0.605, *p* = 0.007 < 0.01), and eco-helping behavior (β = 0.506, *p* = 0.014 < 0.05); thus, H1a, H1c, and H1d are supported. Needs-supplies fit has a significant positive effect on eco-helping behavior (β = 0.375, *p* = 0.049 < 0.05); thus, H2d is supported. Demands-abilities fit has a significant positive effect on task-related green behavior (β = 0.467, *p* = 0.001 < 0.01) and eco-initiatives behavior (β = 0.505, *p* < 0.001); thus, H3a and H3b are supported. However, the person-organization fit has no significant effect on eco-initiatives behavior (β = 0.390, *p* = 0.084 > 0.05); thus, H1b is rejected. Needs-supplies fit has no significant effect on task-related green behavior (β = −0.213, *p* = 0.314 > 0.05), eco-initiatives behavior (β = −0.105, *p* = 0.620 > 0.05), and eco-civic engagement behavior (β = 0.045, *p* = 0.829 > 0.05); thus, H2a, H2b, and H2c are rejected. The demands-abilities fit is not significant for both task-related green behavior (β = 0.129, *p* = 0.362 > 0.05) and eco-initiatives behavior (β = −0.082, *p* = 0.532 > 0.05); thus, H3c and H3d are rejected. The results of the SEM are shown in Figure 2.

### The Moderating Role of Psychological Distance

The second objective of this study is to investigate the possible moderating effects of psychological distance on the relationship between person-organization fit and EGB. We followed the methods and procedures for the analysis of the moderating effect as recommended by scholars (Klein and Moosbrugger, 2000; Kelava et al., 2011). We also used the latent moderated structural equations in the SEM to analyze the potential moderating effects of psychological distance. To this end, we designed a series of models using Mplus7.4. First, we added the concepts of emotional distance and expectation distance; then, we added the direct path of each dimension of the person-organization fit to each dimension of EGB; finally, we made these items, the two dimensions of psychological distance and the three dimensions of the person-organization fit interact (psychological distance × person-organization fit), and tested the influencing path of each interaction item on EGB. The moderation results of emotional distance and expectation distance are shown in Table 6.

**TABLE 6:** Results of psychological distance moderation.

Interaction effect	Estimate	S.E.	Est./S.E.	*P*-Value
VF × EMD→TRGB	0.105	0.060	1.758	0.079
VF × EMD→ECB	0.136	0.057	2.397	0.017*
VF × EMD→EHB	0.154	0.042	3.653	***
NSF × EMD→EHB	0.150	0.039	3.793	***
DAF × EMD→TRGB	0.115	0.067	1.720	0.085
DAF × EMD→EIB	0.048	0.078	−0.610	0.542
VF × EXD→TRGB	0.002	0.079	0.022	0.983
VF × EXD→ECB	−0.079	0.072	−1.091	0.275
VF × EXD→EHB	−0.204	0.043	−4.777	***
NSF × EXD→EHB	−0.176	0.035	−5.085	***
DAF × EXD→TRGB	−0.057	0.093	−0.611	0.541
DAF × EXD→EIB	−0.048	0.078	−0.610	0.542

*p < 0.05, ***p < 0.001. TRGB, Task-related green behavior; EIB, Eco-initiatives behavior; ECB, Eco-civic engagement behavior; EHB, Eco-helping behavior; VF, Values fit; NSF, Needs-supplies fit; DAF, Demands-abilities fit; EMD, Emotional distance; EXD, Expectation distance.

In the results of the moderating effect, the regression coefficient and significance of interaction terms are used to judge whether the moderating effect exists. As shown in Table 6, emotional distance significantly moderates the relationship between values fit and eco-civic engagement behavior (β = 0.136, *p* = 0.017 < 0.05), values fit, and eco-helping behavior (β = 0.154, *p* < 0.001) as well as needs-supplies fit and eco-helping behavior (β = 0.150, *p* < 0.001). Moreover, the effect will be greater when the emotional distance is close rather than distant. Therefore, H4 is partly supported. In addition, the expectation distance significantly moderates the relationship between values fit and eco-helping behavior (β = −0.204, *p* < 0.001) as well as the needs-supplies fit and eco-helping behavior (β = −0.176, *p* < 0.001). However, the effect will be weaker when expectation distance is close rather than distant. So, H5 is rejected.

**FIGURE 3:**
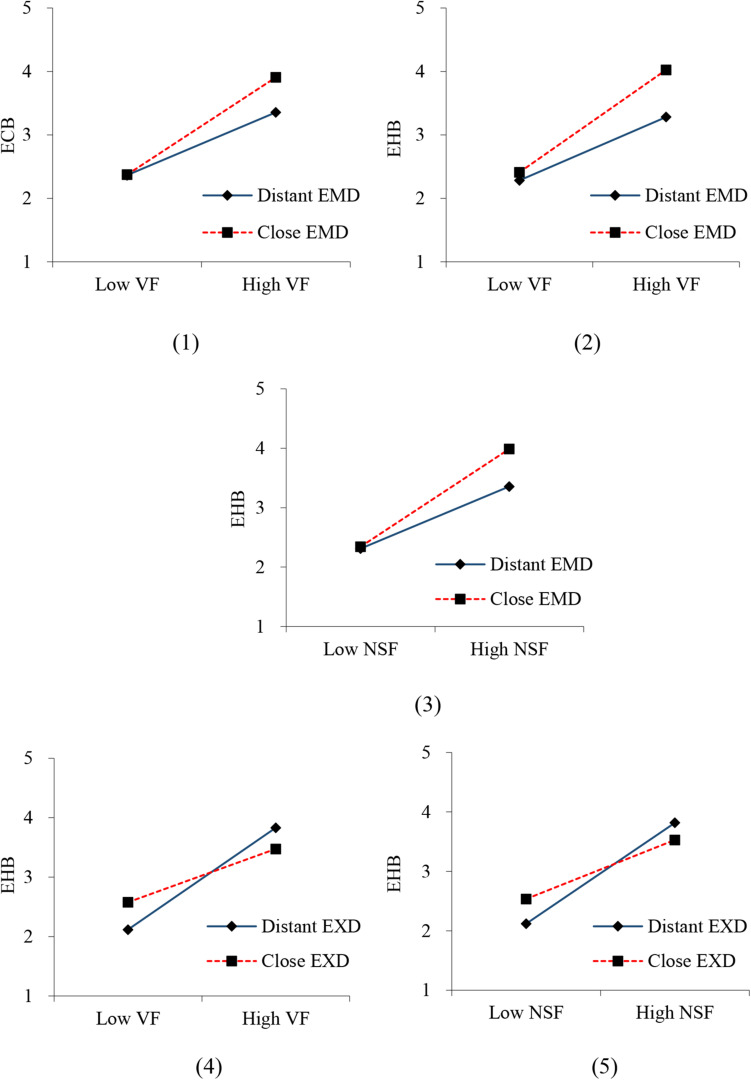
The moderating effect of psychological distance. ECB, Eco-civic engagement behavior; EHB, Eco-helping behavior; VF, Values fit; NSF, Needs-supplies fit; EMD, Emotional distance; EXD, Expectation distance.

According to the results of the moderating effect test, we plotted the simple slope diagram. As Figure 3 shows, in the case of close emotional distance, values fit has a stronger positive effect on eco-civic engagement behavior and eco-helping behavior, and needs-supplies fit also has a stronger positive effect on eco-helping behavior. However, in the case of distant expectation distance, values fit and needs-supplies fit has a stronger positive effect on eco-helping behavior.

## Discussion

This study expands the theory of person-organization fit, introduces psychological distance as a moderating variable, and explores the mechanism of values fit, needs-supplies fit, and demands-abilities fit on EGB. Additionally, it expands existing research from the individual level to the interaction level between individuals and organizations, which provides a new perspective for understanding and promoting EGB, as well as a new path for promoting the green development of enterprises. This study concludes as follows:

(1) Person-organization fit can effectively promote employees’ task-related green behavior and proactive green behavior (eco-initiatives behavior, eco-civic engagement behavior, and eco-helping behavior). The psychological distance between employees and the organization plays a moderating role in this. Managers can increase the degree of person-organization fit by improving recruitment practices and the allocation of human resources in the organization, resulting in higher green performance. They can also promote EGB by changing the psychological distance between employees and the organization.

(2) There are significant differences in the driving effects of the three different types of person-organization fit relationships on the four types of EGB. First, the effect of values fit on EGB is the most significant, and the effect intensity is approximately 0.5, which further verifies that values fit is the most important element of person-organization fit (Chatman, 1991). Second, demands-abilities fit can significantly promote task-related green behavior and eco-initiatives behavior. However, its influence on eco-civic engagement behavior and eco-helping behavior is not significant. Needs-supplies fit only has a positive effect on eco-helping behavior and has no significant effect on the other three types of EGB. Therefore, the three person-organization fits have different effects on the role of different types of green behaviors. Additionally, the roles of the three fits have a certain complementary relationship. Therefore, to promote task-related green behavior and proactive green behavior comprehensively, the values fit, needs-supplies fit, and demands-abilities fit need to be placed together.

(3) Values fit is the most important fit that affects EGB. It has a significant positive effect on eco-civic engagement behavior, task-related green behavior, and eco-helping behavior. When the degree of values fit is high, the employees’ sense of belonging to and identity in the organization will increase and they will actively participate in organizational activities (Hicklenton et al., 2019) and implement more extra-role behaviors (Cable and DeRue, 2002). Thus, values fit significantly promotes employees to actively participate in the organization’s environmental protection activities. At the same time, other studies confirm that values fit positively affects employees’ job satisfaction and organizational commitment to promoting performance behavior (Westerman and Cyr, 2004). The task-related green behavior is already part of work, so the values fit also actively promotes it. In addition, adapting and integrating the values of colleagues within the organization will actively promote relationships among employees, so they are more willing to remind or help colleagues to implement green behavior and work in a more environmentally friendly manner.

However, contrary to our expectations, values fit has no significant effect on eco-initiative behavior. One of the possible reasons for this difference is that this kind of personal behavior reflects more about the personal predisposition of employees (pre-existing values, attitudes, and habits) (Ramus and Killmer, 2007) instead of depending on the degree of values fit between individuals and organizations.

(4) Demands-abilities fit promotes eco-initiatives behavior and task-related green behavior, while needs-supplies fit promotes only eco-helping behavior. When the employee’s ability fits with the work requirements of the organization, employees will have more energy to do other things by choice, such as implementing environmental protection initiatives. Moreover, eco-initiatives behavior is a direct reflection of the environmental protection value orientation of the organization and the awareness it promotes environmental responsibility. In addition, demands-abilities fit has been proven to be an important predictor of employees’ job performance (Lin et al., 2014); thus, this fit will naturally contribute to task-related green behavior. However, demands-abilities fit mainly reflects the relationship between individuals and jobs, rather than the relationship among individuals. Therefore, demands-abilities cannot significantly promote engagement with eco-civic behaviors and eco-helping behavior outside of employees’ duties.

Finally, the result that surprised us most, is that needs-supplies fit only has a positive effect on eco-helping behavior. This result may be due to the complex mechanisms at play in needs-supplies fit and EGB. It is confirmed that higher needs-supplies fit between employees and organizations will bring higher job satisfaction and organizational commitment (Cable and DeRue, 2002; Bahat, 2020). However, it may not directly promote EGB, rather working indirectly through intermediaries such as job satisfaction and organizational commitment. Therefore, it is necessary to study the complex mechanisms at work between needs-supplies fit and EGB further.

(5) Psychological distance has a moderating effect between person-organization fit and EGB, but the moderating effects of emotional distance and expectation distance are opposite to one another. Emotional distance strengthens the relationships between values fit and eco-civic engagement behavior, values fit and eco-helping behavior, as well as needs-supplies fit and eco-helping behavior. This shows that the closer the emotional distance is, the more positive it can promote EGB. This may be because when the emotional connection between employees and the organization is relatively close, it can promote emotional communication, enhance employees’ sense of identity with the organization (Wang et al., 2009), and improve employees’ job satisfaction (Lapierre and Hackett, 2007). This also means that employees may want to perform more behaviors beyond their job responsibilities to improve organizational efficiency (Bowler et al., 2010). So, emotional distance plays a positive role in moderating the relationship between person-organization fit and EGB.

Contrary to our hypothesis, expectation distance weakens the relationship between values fit and eco-helping behavior as well as needs-supplies fit and eco-helping behavior. This may be because expectation distance is a judgment based on interest relationship distance, which emphasizes the acceptance degree of the gap between the benefits obtained by employees in the organization and their own interest goals. The closer this distance cognition based on interest relationship is, the more likely it will make employees’ psychology and behavior more utilitarian. However, eco-helping behavior is a kind of non-utilitarian citizen behavior. Therefore, when the expectation distance is close rather than distant, it will weaken the relationship between person-organization fit and eco-helping behavior. At the same time, employees’ judgment of the expectation distance with regards to their interest relationship may also lead to unhealthy competition among employees within the same organization, thus inhibiting the relationship between person-organization fit and eco-helping behavior.

## Summary and Suggestions

From the perspective of person-organization fit, this study uses the psychological distance between employees and organizations as the moderating variable and explores the impact of different types of person-organization fit on EGB. Through the survey data from 412 employees, the SEM was used to analyze the effects of values fit, needs-supplies fit, and demands-abilities fit on employees’ task-related green behavior and proactive green behavior. In terms of theoretical significance, we expanded the person-organization fit theory, using the psychological distance between employees and organizations as the moderating variable. For the first time, this research studied the effect of person-organization fit on EGB and tested the moderating effect of psychological distance between employees and organizations. The results show that values fit has the greatest effect on EGB, followed by demands-abilities fit. Needs-supplies fit significantly promotes only eco-helping behavior. Psychological distance has a significant moderating effect on the relationship between person-organization fit and EGB. Moreover, the effect of person-organization fit on EGB is enhanced in the case of close emotional distance, while the effect is weakened in the case of close expectation distance. These results provide new insight into understanding employees’ motivation to implement green behavior from the perspective of interactions between individuals and organizations. In addition, it also outlines a new path to promote the green transformation of enterprises.

Our research results provide insights into new ways for enterprise managers to promote green and sustainable behaviors in employees through the practice of green human resource management:

(1) Organizations should improve the degree of fit between individuals and organizations in the process of recruitment and allocation management. First, in the organization’s personnel recruitment and selection process, employees with higher compatibility between personal values and organizational values should be selected as much as possible. Since individual values are stable and do not easily change, enterprises need to choose employees who are more consistent or compatible with the values of the organization and who promote task-related green behavior, eco-civic engagement behavior, and eco-helping behavior. Second, personnel evaluation, training, and performance management are recommended to improve the fit between personal ability and job requirements to promote task-related green behavior and eco-initiatives behavior. Finally, eco-helping behaviors should be promoted through the design of incentive mechanisms (such as salary incentives, training mechanisms, promotion mechanisms, etc.) to improve the fit of needs and supplies.

(2) Organizations should pay attention to the management of employees’ psychological distance, reduce emotional distance, and increase expectation distance. First, more emotional care and human-focused management should be given to employees so that they truly feel valued and part of the organization. Additionally, employees should be encouraged to implement green behavior through the guidance of reasonable expectations and the increase of expectation distance.

In terms of the interactions between individuals and organizations, this research investigated the impact of person-organization fit on EGB, and the results provide an important addition to existing literature on the subject. However, there are still several limitations that need to be noted and can be improved in future research. First, our research is conducted in the context of Chinese culture. In Chinese society where there is a focus on “high context culture,” individual behavior is more likely to be influenced by one’s interaction with an organization. Considering the differences in people’s perception of person-organization fit in different cultures, future research can expand the sample to other cultural backgrounds. Second, only direct paths of person-organization fit and EGB are considered, and indirect paths are excluded. For example, it was found that needs-supplies fit only has a positive effect on eco-helping behavior, while needs-supplies fit simultaneously causes job satisfaction and organizational commitment. Whether these psychological variables can indirectly drive green behavior remains to be tested. Future research could also further explore whether person-organization fit can play an indirect role in EGB through other mediating variables. Third, we used the psychological distance between employees and the organization as the moderating variable, and only two dimensions (emotional distance and expectation distance) from the psychological relationship were selected for moderation. Whether time distance, space distance, and other psychological distances based on real relationships play a role in EGB is worth exploring in future research.

## Data Availability Statement

The raw data supporting the conclusions of this article will be made available by the authors, without undue reservation.

## Ethics Statement

The studies involving human participants were reviewed and approved by the Ethics Committee of China University of Mining and Technology. The participants provided their written informed consent to participate in the study.

## Author Contributions

LM: conceptualization, resources, supervision, project administration, funding acquisition, writing the original draft, writing-review, and editing. YS: methodology, validation, formal analysis, data curation, writing-original draft, writing-review, and editing. XG: investigation, data curation, writing-original draft, writing-review, and editing. HY: visualization, methodology, and writing-original draft. TL: conceptualization, supervision, and investigation. KS: data curation, writing-review, and editing. YQ: conceptualization, investigation, and data curation. ZJ: methodology and data curation. All authors contributed to the article and approved the submitted version.

## Conflict of Interest

The authors declare that the research was conducted in the absence of any commercial or financial relationships that could be construed as a potential conflict of interest.
