# Supplementary material for: Promoting Employee Green Behavior Through the Person-Organization Fit: The Moderating Effect of Psychological Distance
Source: Front Psychol. 2020 Oct 9;11:568385. doi: 10.3389/fpsyg.2020.568385 (PMC7581679; doi:10.3389/fpsyg.2020.568385)
Supplement: Supplementary file 1 [file Data_Sheet_1.docx]

Supplementary Material

**Appendix I**

| **Items** | Ranking | | | | |
| --- | --- | --- | --- | --- | --- |
| **Employee green behavior** | 1 | 2 | 3 | 4 | 5 |
| I can accomplish the environmental protection tasks within my duties competently | Never | Rarely | Sometimes | Often | Always |
| I can fulfill the environmental protection responsibilities clearly specified in the job description |  |  |  |  |  |
| I can accomplish the environmental tasks that the team expects to complete |  |  |  |  |  |
| I can meet the environmental standards of formal work performance requirements |  |  |  |  |  |
| I pay attention to energy conservation and low-carbon travel in my daily work |  |  |  |  |  |
| I pay attention to the secondary use of items in my daily work, such as double-sided printing |  |  |  |  |  |
| I voluntarily carry out environmental actions and initiatives in my daily work |  |  |  |  |  |
| I actively participate in environmental events organized by my company (or department) |  |  |  |  |  |
| I stay informed of my company’s environmental initiatives |  |  |  |  |  |
| I volunteer for projects, jobs or events that address environmental issues in my companies |  |  |  |  |  |
| I am willing to spend time to remind my colleagues to pay attention to environmental protection at work |  |  |  |  |  |
| I spontaneously encourage my colleagues to adopt more environmentally conscious behavior at work |  |  |  |  |  |
| I convince my colleagues to buy environmentally friendly products |  |  |  |  |  |
| I will encourage my colleagues to express their thoughts and opinions on environmental issues |  |  |  |  |  |
| I spontaneously give my time to help my colleagues take the environment into account in everything they do at work |  |  |  |  |  |
| **Person-organization fit** | 1 | 2 | 3 | 4 | 5 |
| The things that I value in life are very similar to the things that my organization values | Strongly disagree | Disagree | Neutral | Agree | Strongly agree |
| My personal values match my organization’s values and culture |  |  |  |  |  |
| My organization’s values and culture provide a good fit with the things that I value in life |  |  |  |  |  |
| There is a good fit between what my job offers me and what I am looking for in a job |  |  |  |  |  |
| The attributes that I look for in a job are fulfilled very well by my present job |  |  |  |  |  |
| The job that I currently hold gives me just about everything that I want from a job |  |  |  |  |  |
| The match is very good between the demands of my job and my personal skills |  |  |  |  |  |
| My abilities and training are a good fit with the requirements of my job |  |  |  |  |  |
| My personal abilities and education provide a good match with the demands that my job places on me |  |  |  |  |  |
| **Psychological distance** | 1 | 2 | 3 | 4 | 5 |
| I will protect organizational interests at the cost of my own interests when necessary | Strongly disagree | Disagree | Neutral | Agree | Strongly agree |
| I feel delight and enjoyment in my organization |  |  |  |  |  |
| The organizational culture of our company gives me a sense of belonging and identity |  |  |  |  |  |
| My role in the organization is in line with my expectations, and my abilities and specialties are being played |  |  |  |  |  |
| I will work harder only if the return of work meets my expectations |  |  |  |  |  |
| If my current job can realize the value of my life, I will actively treat my job |  |  |  |  |  |
